# Learning curve in the endovascular treatment of chronic post-thrombotic syndrome in a French center

**DOI:** 10.1186/s42155-025-00561-y

**Published:** 2025-05-14

**Authors:** Paul Segui, Valérie Monnin-Barès, Monira Nou, Sébastien Bommart, Hamid Zarqane, Juliette Vanoverschelde, Hélène Vernhet-Kovacsik

**Affiliations:** 1 Thoracic and cardiovascular imaging, , Hôpital Arnaud de Villeneuve, 371 Av. du Doyen Gaston Giraud, Montpellier, 34090 France; 2 Vascular Medecine, CHU St Eloi, Montpellier, France

## Abstract

**Purpose:**

Chronic post-thrombotic syndrome (PTS) is a frequent and disabling complication of deep vein thrombosis (DVT) with significant clinical impact. Endovascular stenting (EVS) has established itself as an effective technique but its availability remains limited to expert centers. We sought to identify the key determinants of our learning curve in EVS for PTS and the impact of this experience on our short and long-term results, in order to facilitate territorial dissemination and respond effectively to clinical demand.

**Material and methods:**

We reviewed the records of 68 patients treated in our centre during eight years. We collected patients and disease characteristics, technical elements of the procedure, peri-procedural medical management and detail of the clinical follow-up and imaging.

**Results:**

The median follow-up was 37 months. The primary, primary assisted and secondary patency rates were respectively 74%, 86% and 95%. A clinical benefit was observed in all patients from the start of our activity, without significant change whatever the operator experience. The main determinants of our learning curve were a progressive mastery of the procedure in its technicality and preparation, the evolution of the material and the improvement of the peri procedural management, allowing to reduce the duration of intervention, the rate of endovascular revision (38% to 4%, *p *< 0.01) but also the number of remote thrombotic events (29% to 6%).

**Conclusion:**

EVS appears to be an effective therapeutic option in the management of PTS, with consistent clinical improvement observed even when performed by less experienced operators. Improvement in this technique comes with faster procedures, and a reduction of the occurrence of peri-procedural and long term thrombotic events. The implementation of this type of procedure requires multi-disciplinary collaboration with vascular medicine and corresponding angiologists.

## Introduction

Chronic post-thrombotic syndrome (PTS) is a frequent and disabling complication of deep vein thrombosis (DVT), exposing patients to a significant deterioration in their quality of life [1]. The advent of endovascular stenting (EVS) offers an innovative approach in the management of this pathology, opening new therapeutic perspectives to improve symptoms and prevent long-term complications. This technique is expanding but is still limited to expert centres, with an uneven territorial network.

We reviewed a cohort of patients treated by EVS for PTS, between 2015, the date of initiation of our activity in this field, and 2023. This period represents a significant time window, making it possible to evaluate both the evolution of our practice and the long-term results of the patients treated.

Our results and the evolution of our therapeutic approach are detailed here under a practical prism in order to identify what were the determining axes of our learning curve.

## Materials and methods

### Data collection

A national multicentre database on endovascular treatment of PTS was set up in early 2020, powered by 17 volunteer centres (including ours), initially completed retrospectively (since the beginning of this activity in each of the centres) then prospectively.

Collected data were clinical, technical and radiological, according to the items defined in the Redcap national register [2].

From October 2015 to December 2023, 68 patients underwent venous recanalization for PTS in our centre, including both unilateral iliofemoral and complex iliocaval lesions. The study excluded non-thrombotic obstructive condition (such as May-Turner syndrome).

We subsequently analyzed all procedures recorded in the Picture Archiving and Communication System (PACS) to gain a better understanding of the potential causes of endovascular failure and recovery. A systematic review of pre-, peri-, and post-procedural imaging—both angiographic and CT—was therefore conducted in all patients presenting with early thrombotic recurrence.

The study protocol was IRB approval exempted.

### Procedure

Each patient is evaluated during a dedicated consultation by the operator performing the endovascular procedure, during which all relevant clinical elements are reviewed to determine the indication for treatment.

We use reproducible clinical scores to assess the severity of symptoms and their impact on patients'quality of life. Villalta’s score, classifying the clinical sequelae of the PTS, serves as a reference to assess the severity of symptoms [3], while the Vein Impact Questionnaire (CIVIQ 20) offers a perspective on the quality of life specifically related to venous problems [4].

A direct CT phlebography is systematically performed, enabling detailed analysis of the endovenous lumen to identify areas of occlusion and/or stenosis. This modality also facilitates the detection of more subtle findings, such as endoluminal synechiae, which may not significantly reduce venous diameter but nonetheless contribute to obstructive phenomena [5]. Localization and extent of post-thrombotic venous lesions in the thigh were recorded, and a CT grading for severity was established according to the four-grade scale proposed by Menez et al. [6].

Each indication is validated after multidisciplinary team meeting (including interventional radiologists, angiologists and internists), held monthly in our centre.

All procedures were performed by a senior interventional radiologist (8 years’ experience in interventional radiology at the beginning of the activity), same main operator for all of them, assisted by other interventional radiologists in case of complex procedure, in a dedicated angiography room, using local anesthesia or under general anaesthesia (for iliocaval procedures). The whole radiology team was inexperienced in such procedures before 2015.

Intravenous bolus of 50 UI/kg of unfractionated heparin was injected during the procedure for all patients.

Ultrasound-guided percutaneous access was obtained via the right internal jugular vein in all patients. In cases where retrograde catheterization proved challenging, access was complemented by puncture of the femoral vein on the ipsilateral side of the thrombosis.

In case of iliocaval involvement, a triple access by bilateral femoral and right jugular was systematically used.

Recanalization and stenting were performed according to standards of practice guidelines [7], with the exclusive implementation of Sinus Flex XL (Optimed) or Zilver Flex (Cook Medical) stents [8–10]. Intra Vascular UltraSound (IVUS) is not available in our center and no procedure was performed using this technology.

Final angiography was systematically performed at the end of the procedure. Success criteria were rapid opacification, clearance of intra-stent flow and disappearance of collaterals.

After procedure, anticoagulation treatment included anti-platelet therapy and therapeutic dose of LMWH. Oral anticoagulation was then prescribed for at least 6 months (depending on the patients thrombotic risk factors).

### Results assessment

Immediate technical success was defined as successful recanalization and stent deployment restoring rapid anterograde flow in the targeted vessel.

Duplex ultrasound control was systematic at day one. In case of early thrombosis, a new angioplasty session was reprogrammed within 24 to 36 h depending on the availability of the OR.

Patients were clinically monitored with venous score assessment at 1 month and during follow-up. Stent patency was assessed by ultrasonography at months 1, 6 and 12, then every year thereafter and CT phlebography (direct or indirect) at years 1, 3 and 5 then 5-yearly. Primary patency was defined as confirmed patency of the treated veins on follow-up, without occlusion, or any re-intervention. Primary assisted patency was defined as confirmed patency achieved without occlusion or reintervention, except for preemptive treatment of in-stent stenosis. Secondary patency was defined as established patency after reintervention for stent occlusion.

Intercurring examinations could be performed on clinical call point, or in case of Doppler modification on systematic follow-up (appearance of intrastent thickening with or without stenosis) requiring additional CT imaging.

CT follow-up allowed to analyse stents deployment, occurrence of possible fractures, presence of thickening and/or calcifications, to identify potential risk factors for late thrombosis [11].

The statistical analyses were performed using R software (v. 4.4.3.).

## Results

### Patients

Patients characteristics and PTS baseline lesions are summarized in Tables 1 and 2.

**Table 1:** Patients baseline characteristics

Baseline	Mean (SD)	Median [Q25-75]	min	Max	*n*
Age at treatment	45.7 (16.2)	48.0 [32,0; 57,0]	18.00	76.0	68
Diagnostic-treatment delay (years)	11.8 (12.5)	6.00 [2.00; 17.5]	1.00	41.0	68
Civiq 20 score	60.5 (18.6)	58.5 [44.8; 77.2]	25.0	91.0	60
Villalta score	14.3 (6.00)	13.0 [9.00; 18.0]	4.00	30.0	68
DN4 score	3.67 (2.78)	4.00 [1.00; 6.00]	0	9.00	49

**Table 2:** Baseline characteristics of PTS lesions

PTS baseline lesions		*n* (%)
Type	Cavo bi-iliac	16 (23%)
	Unilateral	52 (77%)
Unilateral lesions side	Left	44 (84%)
	Right	8 (16%)
Post-thrombotic sequelae caudal to the CFV according to the Menez scale	0	28 (41%)
	1	11 (16%)
	2	22 (32%)
	3	7 (10%)

### Technical results and complications

Immediate technical success was achieved in 67 patients (99%). The only recanalization failure in our series occurred early in our experience, before we had access to the dedicated crossing catheter (Cx, Cook Medical) typically used for these procedures.

No procedural-related death occurred during or within months following intervention.

Two grade 3 complications, as per the CIRSE classification [12], were reported. These included a retroperitoneal hematoma requiring intensive care and multiple transfusions, associated with post-thrombotic peri-venous neoangiogenesis and a false passage during recanalization, which occurred in April 2023. Additionally, a complication related to general anesthesia (malignant hyperthermia due to halogenated agents) was reported in May 2023.

Four minor adverse events were also noted (5.9%), related to hematomas at venous puncture points, all occurring after 2018.

Our average follow-up time was 37 months. However, 17 patients were treated in 2023, lowering this follow-up time at the time of data collection.

At the end of the mean follow-up, the primary, primary-assisted and secondary patency were achieved in 73%, 86% and 95% respectively.

### Clinical results

The clinical results are summarized in the Table 3.

**Table 3:** Clinical improvements after intervention

		Baseline (*n* = 68)	Post EVS	Mean Δ	*n*	*P*
Civiq 20, mean (SD)		60.5 (18.6)	33.7 (15.7)	−26.8	57	< 0.001
DN4, mean (SD)		3.67 (2.78)	1.31 (1.51)	−2.36	31	< 0.001
Villalta, mean (SD)		14.3 (6.00)	4.72 (3.98)	−9.60	64	< 0.001
Active Ulcerations		7 (10%)	1 (1.4%)	-	67	< 0.001
Dyspnea	No	19 (28%)	52 (83%)	-	63	< 0.001
	Yes	50 (72%)	11 (17%)	-	-	-

It should be noted that there was no significant change in patients’ clinical scores at inclusion over time, allowing a comparison of the results obtained over the study period.

### Learning curve assessments

**Fig. 1:**
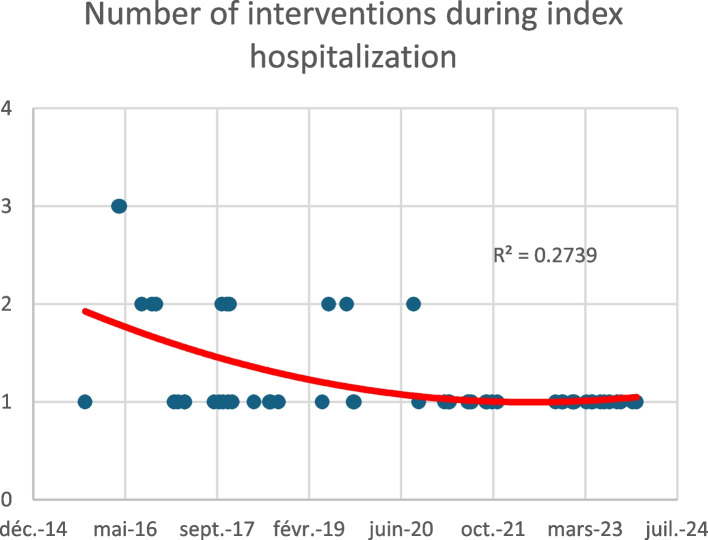
Number of interventions needed to achieve permeability during index hospitalization over time

A major improvement in our care is the reduction of the number of interventions required during index hospitalization, with a decreasing curve towards a single-stage procedure (Fig. 1).

We also observed a significant reduction in the duration of the intervention, as reflected by the fluoroscopy time, particularly for unilateral recanalizations. Intervention time was more variable in cases of bilateral lesions with IVC occlusion, due to the increased complexity of the procedure.

**Fig. 2:**
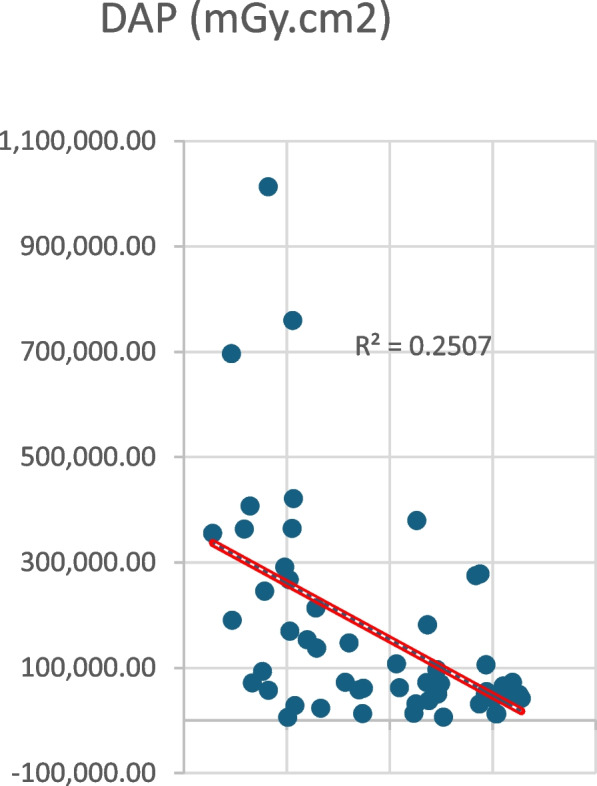
Dose Area Product reduction over time

This reduction in operating time was also accompanied by an even steeper decline in DAP (dose area product) over time (Fig. 2).

We compared the results of our patients before and after the median of our cohort (34 vs 34 patients), in 2020.

We found a significant improvement in the number of treatment sessions, as expected from the curve described above (1.50 vs 1.06, *p *< 0.001). Moreover, we found a significant difference on either side of this median regarding the occurrence of thrombosis (secondary patency or occlusion at the end of follow-up), from 29% before 2020 to less than 6% after (*p *< 0.05).

The main results are presented in Table 4.

**Table 4:** Improvement in patency results before and after median inclusion time

		Before 2020 (*n *= 34)	After 2020 (*n *= 34)	*N*	*p*
Number of interventions for initial treatment, mean (SD)		1.50 (0.615)	1.06 (0.239)	68	< 0.001
Long-term patency (end of follow up)	Primary or primary assisted patency	24 (71%)	32 (94%)	56	0.011
	Secondary patency or occlusion	10 (29%)	2 (6%)	12	-

**Fig. 3:**
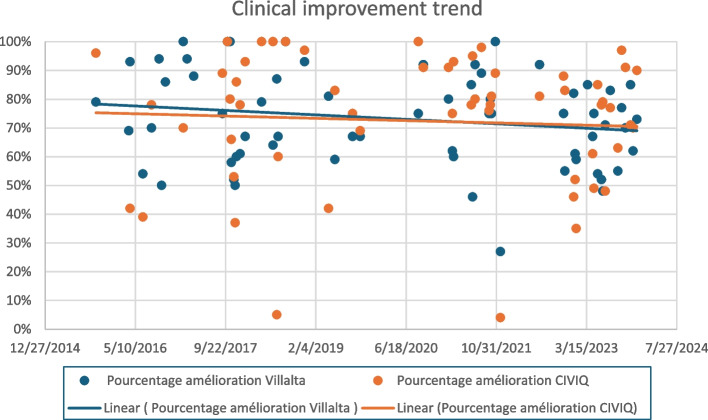
Clinical scores improvement over time

Comparing the clinical results overtime, we found no significant changes in the percentage of improvement in either the Villalta or the CIVIQ scores, with the time curve remaining relatively flat, with a slope close to zero (*R *= 0.06) (Fig. 3).

### Learning curve determinants

We systematically reviewed CT scans and angiography imaging pre-, per- and post-procedure in patients who required multiple sessions during index hospitalisation.

**Fig. 4:**
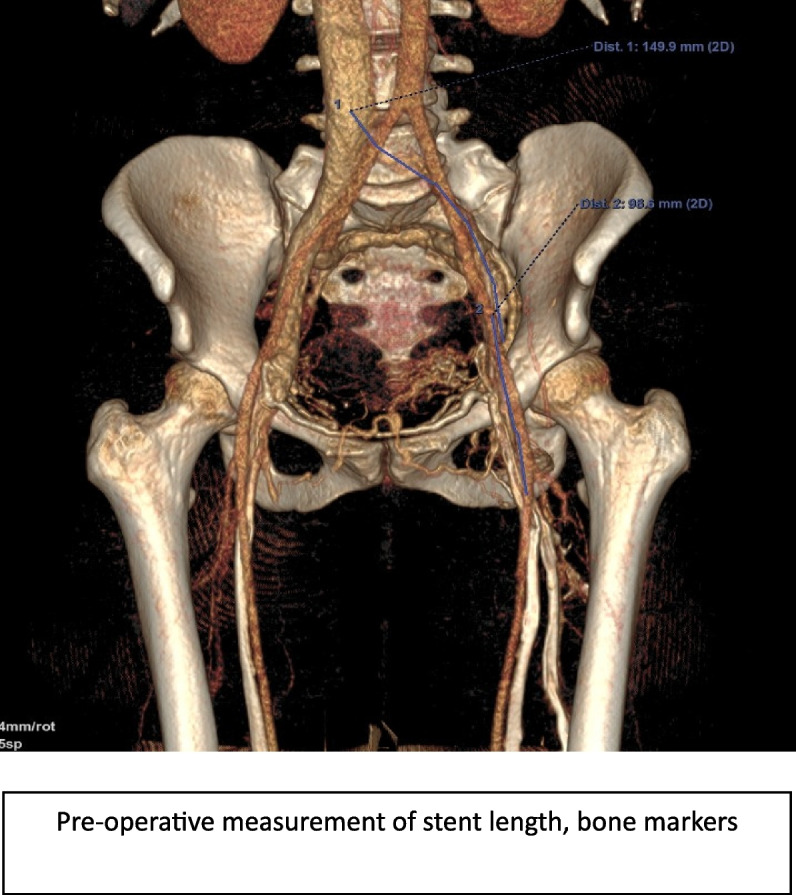
Example of measurements used to chose stenting size and level in EVS procedure

Retrospective analysis of early thrombosis cases highlighted several notable features:

- An oversized balloon in relation to the size of treated vein was observed in 23.5% of cases, possibly suggesting an iatrogenic parietal lesion contributing to thrombosis of the stenting.

- A lack of precision in the stent placement was noted in 64.7% of cases, including a lack of stenosis coverage (*n *= 4), implantation too low compared to lesions resulting in selection of a collateral (SFV or DVP) (*n *= 7), and too distal stenting on spasm (beyond the stenosis) (*n *= 4).

- Hematomas at the puncture site, limiting inflow’s quality, were observed in 11.8% of cases.

- Angiographic success criteria at the end of the procedure were considered suboptimal in 17.6% of cases, with restoration of a washout flow but incomplete disappearance of collaterals (Fig. 4.).

The development of volume-rendered images using direct CT venography has evolved through several stages at our center, despite an identical acquisition protocol. These stages can be divided into three technical phases:

#### Initial period

Volume Rendering Technique (VRT) reconstructions were performed without length measurements, and stent selection was based solely on per-procedural angiographic data.

#### Intermediate period

After several months, reconstructions began incorporating total length measurements of pathological segments.

#### Since late 2018

**Fig. 5:**
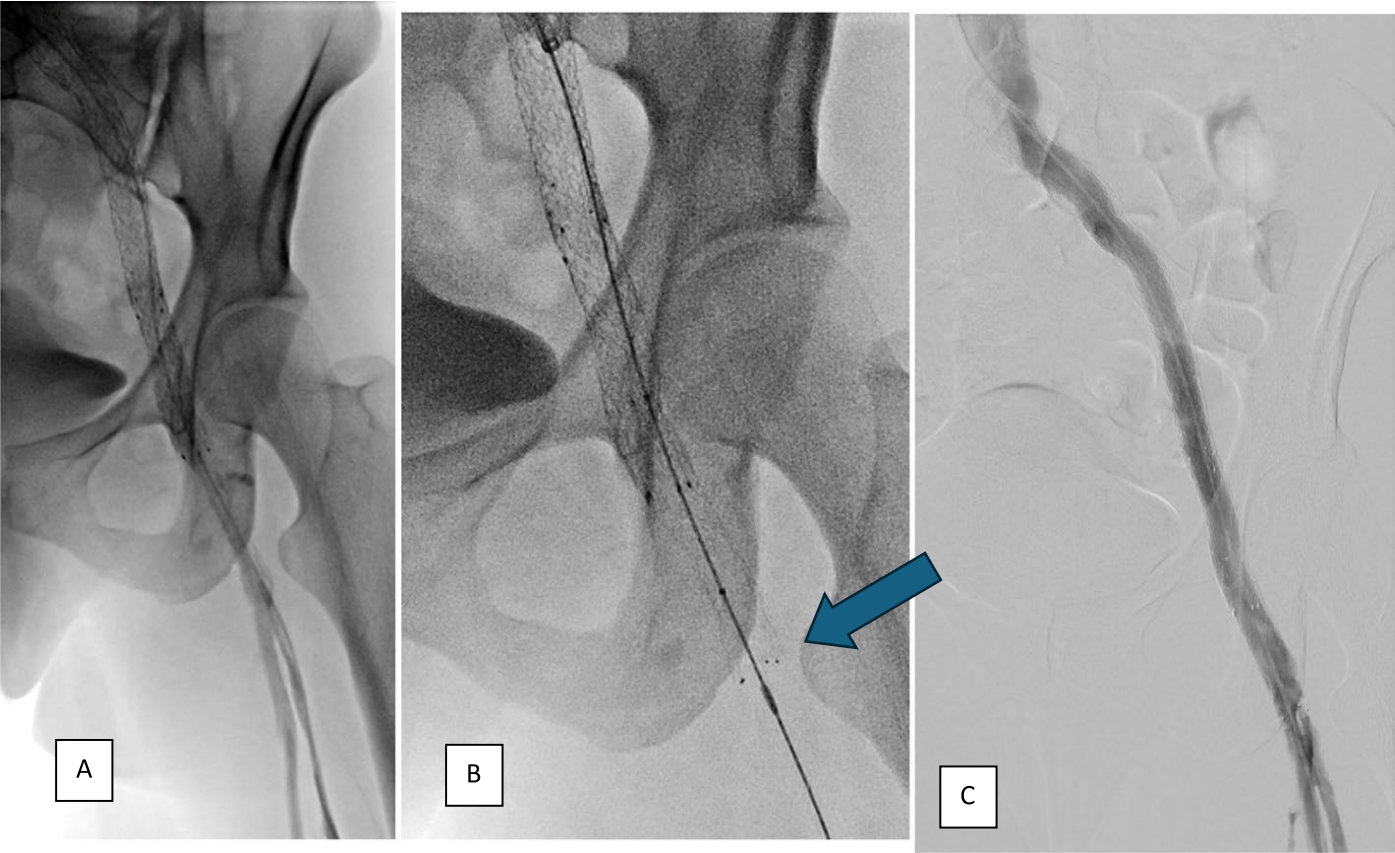
Phlebograms of the same patient at the time of surgery. **A** Initial stenting too short, with phlebography showing disappearance of collaterals, but flow remaining slowed. **B** After comparison with phleboscanner, extension of stenting opposite bone landmarks decided upstream (arrow). **C** Final control phlebography showing rapid washout with disappearance of collaterals

A method incorporating precise measurements adapted to stent lengths was implemented, allowing for pre-procedural stent ordering and accurate visualization of implantation height relative to anatomical landmarks (Fig. 5).

Furthermore, a more detailed examination of native images of the caudal network enabled the identification of synechiae that could potentially affect inflow into the stents, as well as the measurement of affected vein diameters to guide appropriate balloon sizing during angioplasty.

Over time, there has been a significant improvement in the equipment available for venous recanalisation, with the gradual availability of specific tools, to help overcome occlusive lesions.

Significant enhancements have been made to the peri-procedural anticoagulation protocol over time:

#### Initial practice

At the beginning of our activities, low molecular weight heparin (LMWH) was administered for one month, followed by a transition to oral anticoagulants.

#### End of 2021 update

The protocol evolved to use LMWH only during the hospital stay, with a switch to oral anticoagulants upon discharge.

#### Update

Starting in 2023, the first LMWH injection is now administered immediately after the procedure, without waiting for the evening dose.

As our practice has evolved, there has been a noticeable shift towards the use of direct oral anticoagulants (DOACs) instead of vitamin K antagonists (VKAs). DOACs offer easier administration and require less monitoring compared to VKAs, thereby improving patient management and enhancing treatment adherence.

Initially, imaging follow-up was performed by Duplex ultrasound only. Starting in 2020, systematic CT scan follow-ups were introduced with routine checks at 1 year, 3 years, and then every 5 years. This allows for early detection of potential stent stenosis or deformation, enabling timely and effective reintervention before thrombotic events occur. This more rigorous follow-up protocol has been associated with a significant decrease in thrombotic events, from 29 to 6% post-2020, as previously indicated.

Over time, a close collaboration with vascular physicians has been fostered, particularly through the establishment of a monthly Multidisciplinary Team Meeting in 2021. These meetings validate indications for recanalization, discuss complex cases, and address challenges encountered during follow-up. This multidisciplinary approach promotes comprehensive and coordinated patient care, effectively managing risk factors and tailored treatments, thereby reducing the incidence of thrombotic complications. Furthermore, this approach allowed for multiple physicians involvement for each patient, providing broader points of reference in case of complications, each with their own area of expertise.

## Discussion

The initial characteristics of patients in our cohort are broadly consistent with those reported in previous studies on endovascular stenting for PTS [11, 13–16]. However, our cohort includes a notably higher proportion of patients with extensive bilateral iliocaval occlusions. These complex cases, comprising nearly a quarter of our cohort, contrast with the 16% reported in the 2022 national registry [13].

Despite the development of a dedicated PTS care network in our centre, time interval between diagnosis and treatment has not tended to decrease. This finding points to a critical need for continued development and enhancement of PTS management. Effective communication and education targeted at specialists and general practitioners are essential to improving timely diagnosis and intervention.

The comparison of pre- and post-recanalization reveals a significant improvement in patient symptoms and quality of life from the very first cases treated. This"flat temporal curve"of improvement indicates that endovascular stenting is effective even at the onset of practice, regardless of the operator's experience level. It is crucial to note that these positive clinical outcomes were achieved with remarkably low morbidity. Notably, the only two major complications occurred in 2023, one of which related to anaesthesia and not directly linked to the recanalization procedure itself. These outcomes emphasize safety and effectiveness of endovascular recanalization for PTS.

Significant reduction in the number of treatment sessions over time highlights the progress made in mastering the initial procedure. Retrospective review of imaging data shows an improvement over time in the precision of stent placement in healthy areas, better matching of stent and balloon diameters to the treated venous segments, and increased rigor in post-procedural flushing dynamics to ensure complete collateral removal.

Another critical improvement has been the reduction in operative time, reflecting greater procedural efficiency and mastery. The introduction of advanced tri-axial systems and dedicated crossing catheters for venous recanalizations has further contributed to this advancement.

Although IVUS may facilitate optimal landing zone selection, it is not available at our center. Nonetheless, our patency rates are comparable to those reported by centers employing IVUS, which may be reassuring for institutions without access to this modality [7, 13].

Similar trends in procedural learning and outcome optimization have been reported in other interventional radiology fields. For instance, an article on the initiation of prostatic artery embolization (PAE) activities documented immediate clinical benefits for patients despite initially prolonged operative times and significant reductions in fluoroscopy durations over time [17].

This decrease is reflected in our outcomes by the consistently lowered DAP recorded for each intervention. Minimizing radiation is particularly crucial for younger patients. Moreover, since the main area irradiated is the pelvis, reducing exposure is vital for safeguarding long-term health.

Over time, we have shifted towards more efficient anticoagulation strategies, which have significantly contributed to the immediate technical success of procedures and reduced the incidence of early occlusions (Table 5). This underlines the essential role of collaboration with angiologists in improving perioperative management. Thrombotic events during follow-up often occurred under specific circumstances (e.g., cessation of anticoagulation, immobility), and their onset over time varied widely. This variability underlines the necessity of long-term follow-up including and the importance of providing appropriate information to referring physicians and patients themselves. The interventional radiology team must be easily contactable in the event of clinical concern.

**Table 5:** Number of intervention needed to achieve patency according to anticoagulation protocol

	Former protocol (*n *= 42)	New protocol (*n *= 26)	*N*	*p*
Single intervention	62% (26)	96% (25)	51	** < **0.01
> 1 intervention before hospital discharge	38% (16)	4% (1)	17	-

The introduction of multidisciplinary team meetings with vascular physicians has enabled more rigorous follow-up protocols to be drawn up, as well as collegial discussion of complex cases in order to establish the correct indications for endovascular treatment.

Several limitations should be acknowledged in this study. The retrospective and single-center nature of our cohort, coupled with the focus on the progression of a single operator, may constrain the generalizability of our findings, as they may not fully reflect outcomes in other clinical settings or operators. Further prospective, multicenter studies are warranted to confirm these results and better assess reproducibility across various practice environments. Furthermore, the observed reduction in distant thrombotic events over time must be interpreted with caution, as it is influenced by the decreasing follow-up duration for the more recently treated patients. Despite this, our average follow-up period remains substantial (37 months). Additionally, the relatively shorter follow-up for post-2020 patients may affect outcome comparisons.

## Conclusion

This study suggests that an operator can perform endovascular recanalization for PTS safely with convincing results from the start of his experience. These procedures remain technically challenging, requiring learning curve to reduce time intervention and thrombotic recurrence rates in short or long term. Close collaboration with vascular medicine and referring physicians, and the creation of a genuine care pathway, help to improve management of these patients.

These positive results emphasized the importance of sharing this local experience to other centres planning to develop similar activities in interventional radiology activities.

## Data Availability

The datasets used and/or analysed during the current study are available from the corresponding author on reasonable request.

## Abbreviations

PTS: Post-Thrombotic Syndrome
DVT: Deep Vein Thrombosis
EVS: Endovascular stenting
VKA: Vitamin K Antagonist
LMWH: Low Molecular Weight Heparin
DOAC: Direct Oral AntiCoagulant
DAP: Dose Area Product.
CT: Computed Tomography
VRT: Volume Rendering Technique
SFV: Superficial Femoral Vein
DFV: Deep Femoral Vein
CFV: Common Femoral Vein
IVUS: Intra Vascular Ultra Sound
